# Two Cases of Post-partum Hemorrhage

**Published:** 1894-10

**Authors:** A. S. Fuller

**Affiliations:** Resident Physician St. Joseph’s Infirmary, Houston


TEXAS MEDICAL JOURNAL.

ESTABLISHED JULY, 1885.

PUBLISHED MONTHLY.—SUBSCRIPTION $2.00 A YEAR.

VOL. X. AUSTIN, OCTOBER, 1894. NO. 4.

### Original Contributions.

For Texas Medical Journal.

### TWO CASES OF POST-PARTUM HEMORRHAGE.

BY A. S. FULLER, M. D., RESIDENT PHYSICIAN ST. JOSEPH'S INFIRMARY, HOUSTON.

[Read before Houston District Medical Society.]

IN CHOOSING these two cases as the subject of to-night's paper, I had no intention of alluding to those cases in which the hemorrhage is soon checked by the simple methods mentioned in every text-book, but rather of referring to those alarming, but happily rare cases in which, in spite of the efforts of the physician, the hemorrhage is so severe and persistent as to result in the collapse of the patient; and of calling your attention to a useful, and, as I think, not sufficiently well known point in the treatment of such a condition of collapse, be it due to either post-partum, or any other form of hemorrhage.

Such cases are happily few and far between, and possibly their very rarity makes them a greater source of anxiety to us when they do occur.

It was my evil fortune about five years ago to meet with two such cases, both of which nearly resulted in the death of the patient, and which, I am sure, would have ended fatally but for the method of treatment adopted to restore the patient after all the methods usually employed had failed. This method consists of injections of a weak hot solution of sodium chloride into the peritoneal cavity, a proceeding which I believe to be devoid of all danger, and almost as rapidly effective as transfusion or intravenous saline injections. The history of the cases is as follows:

No. 1.—Patient, age about 30, was easily delivered. A few moments after delivery expulsion of the placenta took place, and hemorrhage commenced. It came with a gush, deluging the bed clothes and reaching the floor. The uterus would not contract. Ergot was given by the mouth and ergotine hypodermically, and pressure through the abdominal walls made by grasping the fundus. These methods being unsuccessful in causing contraction, clots were removed by the insertion of the hand into the uterus, and injections of hot water used. At length, with much difficulty, I got the hemorrhage checked, but not before the patient was collapsed; pulse very rapid and hardly perceptible; respiration, sighing, and rather rapid; face and lips bloodless, and whole surface of body covered with a cold, clammy perspiration. Stimulants were given hypodermically, patient's head lowered, and extremities raised, and bottles of hot water were applied. These proceedings failing to produce reaction, I injected about 1½ pints of a hot 2 per cent. solution of sodium chloride into the peritoneal cavity, using a trocar and canula attached to an ordinary fountain syringe. The result was remarkable. In a short time the pulse gained in strength and diminished in frequency, the surface of the body became warm, and the patient made a good recovery. I stayed with her about three hours, and was then able to leave, feeling easy in my mind.

No. 2.—Patient, age 27; history of flooding in two out of five previous labors. Pulse was about 100, an hour after the placenta was expelled, so I decided not to leave the house; about an hour later the uterus relaxed and flooding commenced. The hemorrhage was only arrested by swabbing with tinct. ferri perchlor. and water, of the strength of 1–5, after all other methods had failed. The patient was then pulseless, surface cold and clammy, and extremely pale. Stimulants were given hypodermically, with no effect; lowering of the head and elevation of the extremities brought no response. I injected about two pints of hot salt solution, 2 per cent., into the peritoneal çavity with extremely gratifying results. Her condition improved considerably in a few minutes, and she progressed steadily until in about six hours I was able to leave her.

Both these cases made a good recovery. In both cases improvement was noticable in a few minutes, and in neither case were there symptoms of peritonitis or other evil effects attributable to the injections.

This proceeding was suggested to me by the effect produced by rectal saline injections. Noticing their effect caused me to wonder whether the same amount of stimulation and absorption could not be produced more certainly and rapidly by the use of intra-peritoneal injections. This, combined with the effect of flushing the abdominal cavity with hot water during the performance of laparotomy decided me on trying them if ever I should meet with cases in which they might be useful. It is, of course, only very rarely that we meet with cases which are sufficiently serious to require the addition of some liquid to the circulation, in order to restore the patient, but in such cases, whether the cause of the collapse is post-partum, or any other form of hemorrhage, I believe intra-peritoneal injections to be quite as effectual as transfusion or intra-venous saline injections, over both of which they possess the following advantage: simplicity and rapidity of execution, the avoidance of the entrance of air into the circulation, and of special additions to the obstetric or emergency bag.

I think there is no doubt as to its being quicker and more simple to thrust a trocar and canula into the peritoneal cavity than to open a vein and manipulate a more or less intricate arrangement of tubes and valves, as described in the tex-book accounts of transfusions, which, however, I have never seen performed. It is certainly a proceeding with which the practitioner is more familiar, for there are few who have not performed the operation of tapping a patient for ascites, which is only this proceeding reversed. The entrance of air into the veins, and thence to the heart, is of course an impossibility, which cannot be said of intravenous injection, though of course it may be avoided in them with reasonable care. The only necessary additions to the obstetric or emergency bag are a trocar and canula, an addition which is neither expensive nor cumbrous. In my eyes, however, the chief value of this proceeding is the simplicity and rapidity of execution, for there are no cases in which simplicity is a greater boon to the practitioner, and rapidity of more value to the patient.

There are, of course, objections to this proceeding, and the first which will be mentioned is the risk of peritonitis. This will depend greatly on the state in which the physician keeps his instruments. If they are kept in that state of cleanliness which a due regard for his patients' safety and his own reputation will lead every one to use, and only water which has been boiled be used, I believe the risk of peritonitis to be exceedingly small, particularly in a patient as thoroughly depleted as these patients must be, for such depletion in itself must militate against any extensive inflammation taking place, besides causing the fluid to be absorbed into the system rapidly, and so leaving no foreign substance in the peritoneal cavity to serve as an irritant and so cause inflammation to take place. The next objection is the risk of puncture of the intestine. I do not think there is much danger of this occurrence with the ordinary care, and even if it do take place, with a small or medium sized instrument the effects of such a puncture are not serious. This objection will, however, bring up one class of cases of collapse due to hemorrhage in which this proceeding should *not* be used: cases of hemorrhage from typhoid ulcers. In these cases the abdomen is so flattened and retracted that it is difficult not to puncture the intestines, and their irritability is so great that they will not retain the solution long enough to allow of its absorption, and its irritation will probably bring on another hemorrhage and so lessen the little chance the patient still has. These are the chief objections to the method, and they are in my opinion more theoretical than practical. But granting that they do exist in their fullest force, are we justified in looking at probable risks in the face of the pressing and immediate danger that exists in these cases? The manner in which intra-peritoneal injections act, is, I believe, essentially the same as that in which transfusion or intra-venous saline injections act, viz: by adding to the system fluid which is carried into the circulation, giving the heart something to pump on and so stimulating its action. That fluids are easily absorbed by the serous surface of the peritoneum, and under adverse circumstances too, is often seen in the effect produced by hydragogue purgatives in the treatment of ascites. If absorption takes place under such circumstances, how rapidly should we expect it to occur in cases in which the whole system is crying for fluid and the ordinary equilibrium of the blood pressure is so disturbed as to greatly favor absorption? In addition to this supply to the system their action is probably increased by the stimulating effect of heat applied almost directly to the heart. We all use heat for its stimulating effect, in the shape of hot water bottles, etc., but these methods certainly cannot act as rapidly as heat applied directly in the peritoneal cavity, where every particle is used in the body, instead of a large part being lost by radiation into the external atmosphere.

I am sure, however, whatever may be their method of action, that intra-peritoneal injections do produce a rapid and excellent effect, and while I would not recommend them in any cases of hemorrhage except those in which the patient fails to respond to simpler methods of treatment, I feel sure that any one who tries them in such cases will not be disappointed.

My chief reason for choosing these two cases and this method of treatment as the subject of to-night's paper, is that the literature on the subject is so slight that I have only been able to find reports of four cases in which it was tried, and those reports only mention the bare fact that it was tried, and give no details as to results, etc. This must be my excuse for taking up your time with the description of a proceeding of which the best I can say is that I hope we shall none of us in future ever meet with cases in which its use may be indicated.

